# Effect of Nickel Stress on Nitrogen Metabolism in Cucumber Plants

**DOI:** 10.3390/ijms26199327

**Published:** 2025-09-24

**Authors:** Ewa Gajewska, Aleksandra Witusińska

**Affiliations:** Department of Plant Physiology and Biochemistry, Faculty of Biology and Environmental Protection, University of Lodz, Banacha 12/16, 90-237 Łódź, Poland; aleksandra.witusinska@biol.uni.lodz.pl

**Keywords:** glutamate, nickel stress, nitrogen assimilation, non-protein thiols, proline

## Abstract

Excessive concentrations of nickel (Ni) are phytotoxic, leading to disturbances in plant cell structure and function. Although some attempts have been made to elucidate the Ni impact on plant metabolism, the effect of this metal on nitrogen assimilation and transformation of nitrogen compounds still remains poorly understood. The objective of our study was to gain a better insight into the Ni influence on nitrogen metabolism in cucumber plants. Nitrogen metabolism-related enzyme activities and selected metabolite contents were assayed using spectrophotometric methods. Additionally, in the leaves, nitrogen assimilation-involved gene expression was analyzed using quantitative real-time PCR. Nickel treatment resulted in a decline in ${\mathrm{NO}}_{3}^{-}$ content in the leaf and ${\mathrm{NH}}_{4}^{+}$ content in the root. In the leaf, ferredoxin-dependent glutamate synthase (Fd-GOGAT) activity decreased, while NADH-dependent glutamate synthase (NADH-GOGAT) and glutamate dehydrogenase (GDH) activities increased. The GDH activity showed increases in both its aminating (NADH-GDH) and deaminating (NAD-GDH) functions. The activities of the other enzymes involved in nitrogen assimilation were not influenced by Ni stress. In the root, the activities of most enzymes were downregulated by Ni treatment except for NADH-GDH and NAD-GDH activities which showed increases. While glutamate content remained unaltered after Ni exposure in the leaf, in the root it was slightly lowered. In contrast to the leaf, showing accumulation of non-protein thiols and proline, in the root, these compound contents were markedly decreased. Our study revealed an organ-specific response of cucumber plants to Ni treatment. Accumulation of glutamate derivatives involved in response to heavy metal stress without significant changes in glutamate content may suggest that in the leaf, the induction of NADH-GOGAT and NADH-GDH activities efficiently compensates for the reduced Fd-GOGAT activity. Additionally, the increased NADP-ICDH activity may support glutamate production by providing 2-oxoglutarate for reactions catalyzed by NADH-GOGAT and NADH-GDH.

## 1. Introduction

Nickel, although classified as an essential plant nutrient, becomes toxic to most plant species at excessive concentrations. The detrimental effect of this heavy metal on plants is manifested by growth inhibition and may result in a significant deterioration in the yield quantity and quality of crop plants. Nickel phytotoxicity has been attributed, among others, to its ability to interfere with various physiological and metabolic processes. Although attempts have been made to explore the influence of Ni on the plant metabolism, the mechanisms of its impact on the nitrogen (N) metabolism remain poorly understood [1,2,3]. Nitrogen is one of the most important elements determining plant development and crop yield. Nitrate ion (${\mathrm{NO}}_{3}^{-}$) is the most available N source for higher plants. After being taken up by plants, ${\mathrm{NO}}_{3}^{-}$ is reduced to ammonium ion (${\mathrm{NH}}_{4}^{+}$) by the sequential action of nitrate reductase (NR, EC 1.6.6.1) and nitrite reductase (NiR, EC 1.7.7.1). Nitrate reduction occurs in both the roots and aboveground parts of the plant; however, it is spatially separated between the cytosol (for NR) and chloroplasts/plastids (for NiR) within the cells. Firstly, cytosolic NR reduces ${\mathrm{NO}}_{3}^{-}$ to nitrite ion (${\mathrm{NO}}_{2}^{-}$), which is subsequently translocated to chloroplasts, or plastids in non-photosynthesizing organs, and reduced by NiR to ${\mathrm{NH}}_{4}^{+}$ [4,5]. Incorporation of ammonium into carbon skeletons occurs mainly via the combined action of glutamine synthetase (GS, EC 6.3.1.2) and glutamate synthase (GOGAT). GS catalyzes the ATP-dependent amination of glutamate to form glutamine, while GOGAT mediates the reductive transfer of glutamine’s amide nitrogen to 2-oxoglutarate, yielding two molecules of glutamate [6,7]. Two GS isozymes have been identified in plants: the cytosolic (GS1) and the chloroplastic/plastidic (GS2) [8,9]. Based on the electron donor used, GOGAT is classified in two forms: ferredoxin-dependent (Fd-GOGAT, EC 1.4.7.1) and NADH-dependent (NADH-GOGAT, EC 1.4.1.14). While in the leaves, GS2 and Fd-GOGAT are predominant, while in the roots GS1 and NADH-GOGAT are the most abundant and mainly responsible for the primary ${\mathrm{NH}}_{4}^{+}$ assimilation [4]. In addition to the GS-GOGAT cycle, glutamate dehydrogenase (GDH, EC 1.4.1.2) localized in mitochondria and, in smaller amounts, in chloroplasts, is able to incorporate ${\mathrm{NH}}_{4}^{+}$ into organic compounds. It catalyzes the reversible NADH-dependent amination of 2-oxoglutarate, resulting in the production of glutamate [10]. Due to its lower affinity for ${\mathrm{NH}}_{4}^{+}$, as compared to GS, GDH seems to play a minor or negligible role in the primary ${\mathrm{NH}}_{4}^{+}$ assimilation and is believed to participate mainly in glutamate catabolism, providing carbon skeletons for the tricarboxylic acid cycle [11]. However, it is suggested that under stress, when GS-GOGAT is not fully operative, GDH may be responsible for the maintenance of the internal glutamate concentration [12]. Aminotransferases are considered to be other enzymes involved in controlling the glutamate level in plant tissues. Glutamate is formed by the transfer of the α-amino group from alanine or aspartic acid to 2-oxoglutarate by alanine aminotransferase (AlaAT, EC 2.6.1.2) or aspartate aminotransferase (AspAT, EC 2.6.1.1), respectively. Since reactions catalyzed by AlaAT and AspAT are reversible, glutamate may also serve as an amino donor for the amination of pyruvate and oxalate, resulting in the production of alanine and aspartic acid, respectively [13]. Apart from donating amino groups for the production of other amino acids in transamination reactions, glutamate is used as a substrate for the synthesis of various N-containing compounds, including those involved in plant response to heavy metal stress, such as non-protein thiols and proline.

Nitrogen metabolism is tightly linked to carbon metabolism, which provides carbon skeletons required for N assimilation into organic compounds. The key role in the coordination of these two metabolic pathways is ascribed to cytosolic isocitrate dehydrogenase (NADP-ICDH, EC 1.1.1.42), which catalyzes the oxidative decarboxylation of isocitrate to 2-oxoglutarate. Apart from supplying a substrate for glutamate production, NADP-ICDH is considered to be an important source of NADPH, a principal reductant responsible for redox homeostasis in plant cells [14,15].

Cucumber (*Cucumis sativus* L.), one of the most important vegetable crops worldwide, is considered to be a species of increased N dependence [16]. Our previous studies revealed that the cucumber cv. Cezar exhibited a relatively high sensitivity to Ni, showing about 50% growth reduction and severe symptoms of toxicity on the leaves after treatment with 10 μM Ni. In comparison, wheat seedlings used in our earlier experiments showed a similar degree of growth inhibition at the dose of 100 μM Ni [17,18]. Compared to other heavy metals, relatively little research has been devoted to the influence of Ni on plant nitrogen metabolism, and the reported results are contradictory. Therefore, the objective of our study was to gain a better insight into the Ni effect on nitrogen metabolism in cucumber plants. In addition to the enzyme activities involved in the primary N assimilation, their substrate and product contents, the N-metabolism derived metabolites, namely non-protein thiol and free proline levels, were also investigated.

## 2. Results

### 2.1. Growth and Ni Accumulation

Nickel treatment resulted in about a 50% decrease in the fresh weight of the cucumber leaf and root. Compared to the leaf, the root growth was slightly more affected. The accumulation of Ni in the root was about 2-fold higher than in the leaf. Extending the treatment time did not result in a greater reduction in growth and Ni accumulation in cucumber plant tissues (Table 1).

### 2.2.$\;{NO}_{3}^{-}$ and ${NH}_{4}^{+}$ Contents and NR and NiR Activity

**Figure 1:**
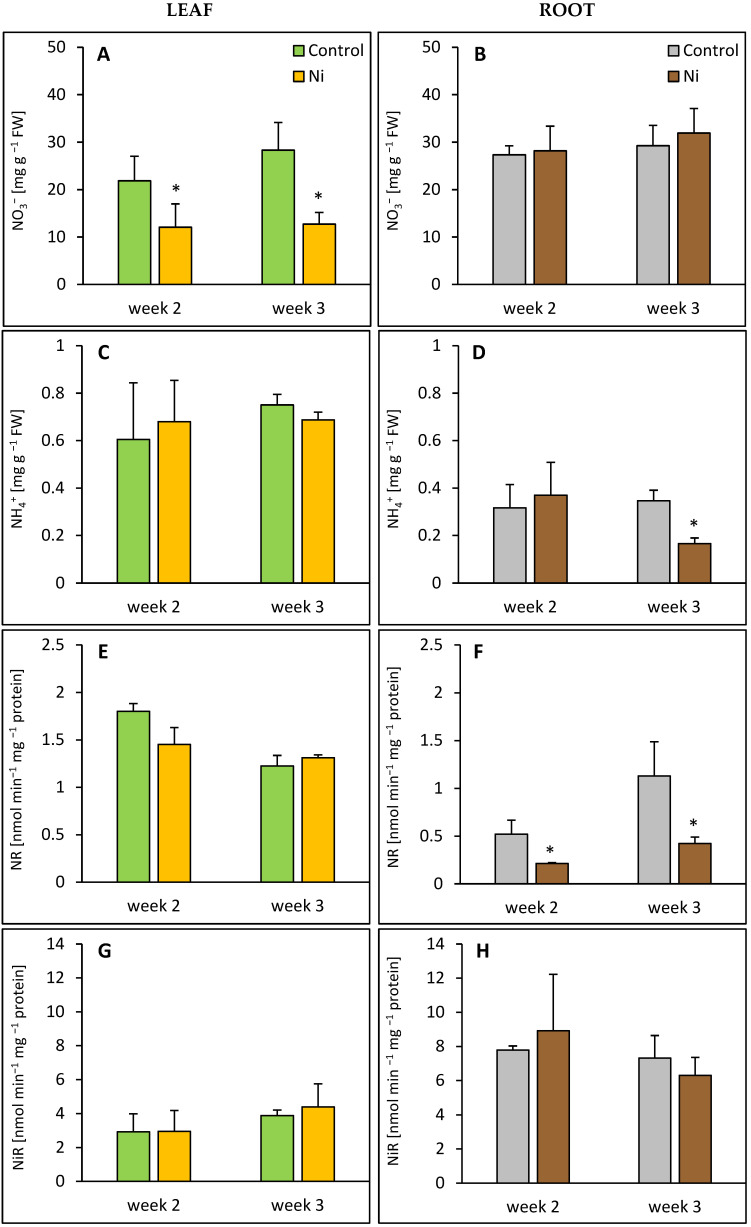
Effect of Ni treatment on ${\mathrm{NO}}_{3}^{-}$, ${\mathrm{NH}}_{4}^{+}$ contents in cucumber leaf (**A**,**C**) and root (**B**,**D**) and NR, NiR activities in cucumber leaf (**E**,**G**) and root (**F**,**H**). Data represent mean values ± SD (*n* = 3–5); * indicates values that differ significantly from the control at *p* ˂ 0.05.

Exposure of cucumber plants to Ni by 2 and 3 weeks lowered the leaf ${\mathrm{NO}}_{3}^{-}$ content by 45% and 51%, respectively (Figure 1A), while in the root, the ${\mathrm{NO}}_{3}^{-}$ level remained unaffected (Figure 1B). No significant changes in ${\mathrm{NH}}_{4}^{+}$ content were observed in the leaf (Figure 1C); however, in the root it was lowered by about 50% after a 3-week treatment (Figure 1D). While in the leaf, NR activity was not altered under conditions of Ni stress (Figure 1E), meanwhile in the root it was markedly inhibited, by 59% and 63%, after 2 and 3 weeks, respectively (Figure 1F). Both the leaf and the root did not show significant alterations of NiR activity after Ni application (Figure 1G,H).

### 2.3. GS and GOGAT Activities

**Figure 2:**
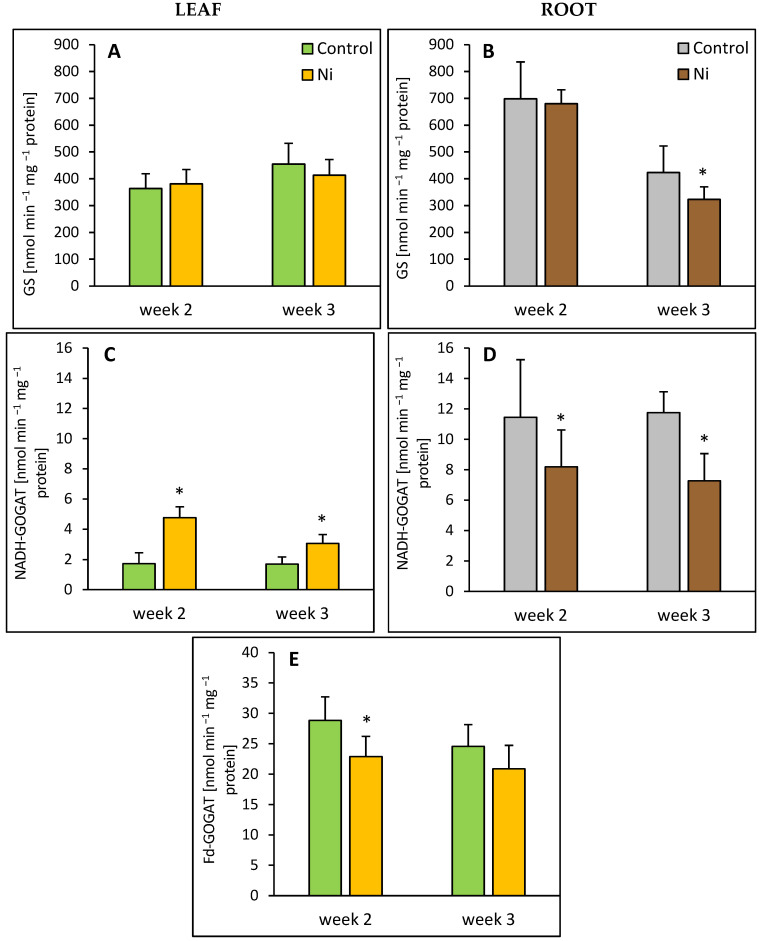
Effect of Ni treatment on GS, NADH-GOGAT, and Fd-GOGAT activities in cucumber leaf (**A**,**C**,**E**) and root (**B**,**D**). Data represent mean values ± SD (*n* = 4–7); * indicates values that differ significantly from the control at *p* ˂ 0.05.

Treatment of cucumber plants with Ni did not affect GS activity in the leaf (Figure 1A); however, in the root, after 3 weeks of the metal exposure, about a 25% decrease in GS activity was observed (Figure 2B). The activity of Fd-GOGAT, the dominating GOGAT form in the leaves, decreased by 21% in response to Ni application (Figure 2E). In contrast, the activity of NADH-GOGAT, accounting for about 6% of the total leaf GOGAT activity showed about 2.8-fold and 1.8-fold enhancement in response to Ni treatment (Figure 2C). Contrary to the leaf, NADH-GOGAT activity in the root was lowered by 28% and 39%, after 2 and 3 weeks of Ni treatment, respectively (Figure 2D).

### 2.4. GDH and Aminotransferase Activities

**Figure 3:**
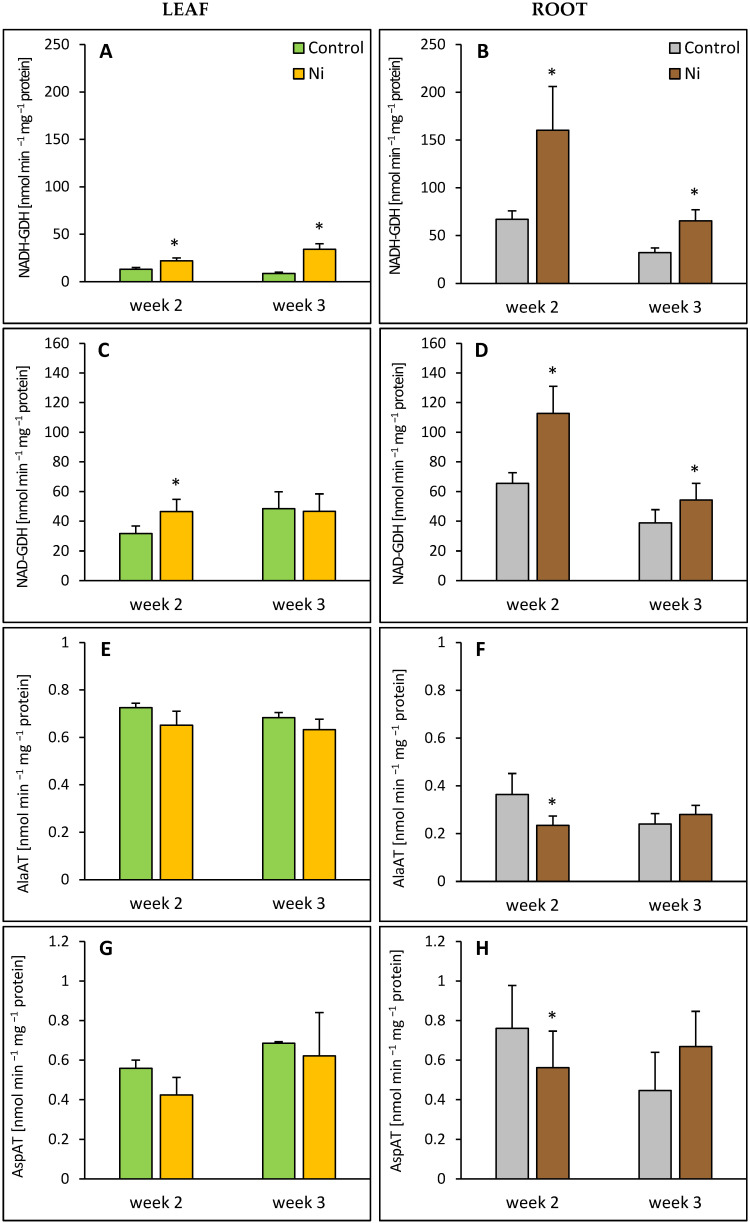
Effect of Ni treatment on NADH-GDH, NAD-GDH, AlaAT, and AspAT activities in cucumber leaf (**A**,**C**,**E**,**G**) and root (**B**,**D**,**F**,**H**). Data represent mean values ± SD (*n* = 4–7); * indicates values that differ significantly from the control at *p* ˂ 0.05.

GDH activity was measured in both the aminating (NADH-GDH) and deaminating (NAD-GDH) directions. Both the leaf and the root responded to Ni treatment with an increased NADH-GDH activity. In the leaves of the Ni-treated plants, the activity of this enzyme was about 1.7-fold and 4-fold higher than in the control after 2 and 3 weeks, respectively (Figure 3A). In the root, increases in NADH-GDH activity were less pronounced, up to 140% and 100% over the control level after 2 and 3 weeks, respectively (Figure 3B). Similarly, NAD-GDH activity was elevated in both organs of the Ni-treated cucumber plants. While in the leaf, a significant increase in NAD-GDH activity, 47% above the control, was found only after 2 weeks; in the root, the activity of this enzyme was enhanced after 2 and 3 weeks, by 72% and 40%, respectively (Figure 3C,D). In the root, 2 weeks after Ni treatment, AlaAT and AspAT activities exhibited decreases, by 36% and 26%, respectively (Figure 3F,H). In contrast, the leaf aminotransferase activities were unaffected by Ni (Figure 3E,G).

### 2.5. Non-Protein Thiol, Proline, Glutamate, and Total Protein Contents

**Figure 4:**
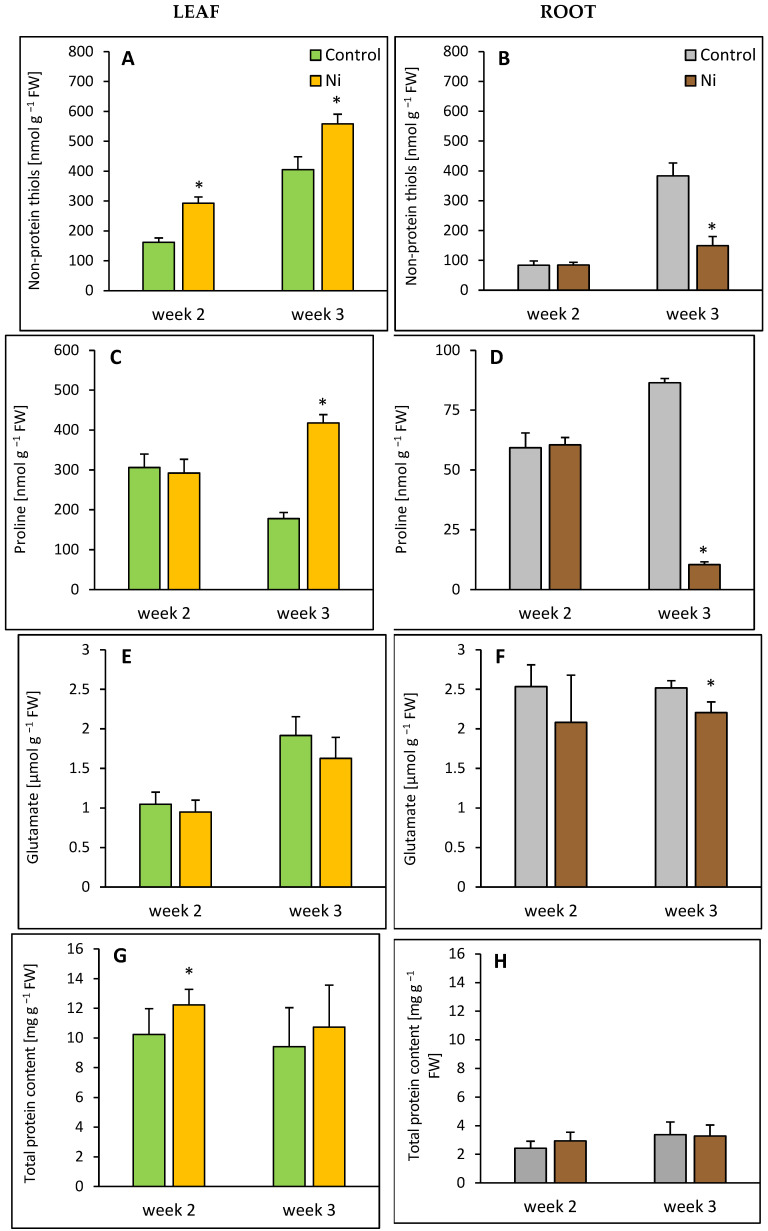
Effect of Ni treatment on non-protein thiols, proline, glutamate, and total protein contents in cucumber leaf (**A**,**C**,**E**,**G**) and root (**B**,**D**,**F**,**H**). Data represent mean values ± SD (*n* = 3–7); * indicates values that differ significantly from the control at *p* ˂ 0.05.

In the leaf, after 2 and 3 weeks of Ni exposure, an increase in non-protein thiol content was found, by 81% and 37% over the control level, respectively (Figure 4A). A significant accumulation of proline (almost 2.5-fold compared to the control) was found after 3 weeks (Figure 4C). At the same time, in the root, a decline in proline and non-protein thiol contents was observed, by 88% (Figure 4D) and 61% (Figure 4B), respectively.

While in the leaf, glutamate content was not significantly influenced by Ni treatment (Figure 4E), meanwhile, in the root, it was slightly decreased, by 12% after 3 weeks (Figure 4F). Total protein content increased in the leaf after 2 weeks of Ni exposure, by 19% compared to the control, but in the root, it remained unaltered (Figure 4G,H).

### 2.6. NADP-ICDH Activity

**Figure 5:**
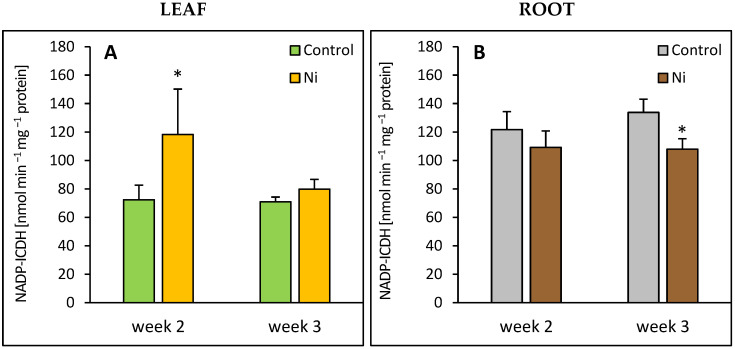
Effect of Ni treatment on NADP-ICDH activity in cucumber leaf (**A**) and root (**B**). Data represent mean values ± SD (*n* = 4); * indicates values that differ significantly from the control at *p* ˂ 0.05.

In the leaf, an increase in NADP-ICDH occurred after 2 weeks of the metal exposure, by 63% over the control level (Figure 5A). In the root at the end of the experiment, the activity of this enzyme was lowered by 20%.

**Figure 6:**
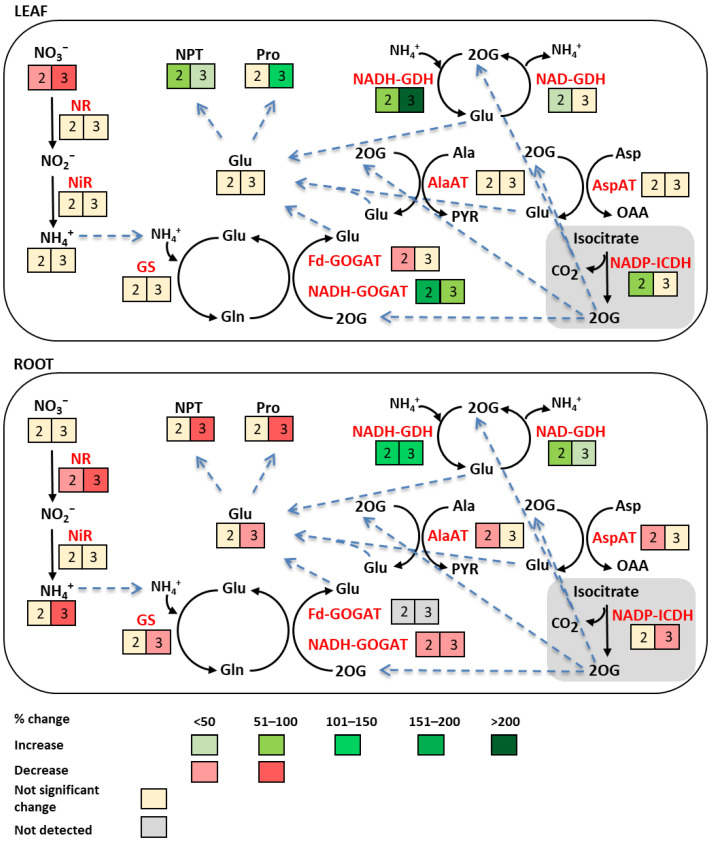
Schematic representation of N metabolism changes in cucumber leaf and root under Ni stress. Glu—glutamate, Gln—glutamine, 2OG—2-oxoglutarate, NR—nitrate reductase, NiR—nitrite reductase, GS—glutamine synthetase, GOGAT—glutamate synthase, GDH—glutamate dehydrogenase, Ala—alanine, Asp—aspartate, PYR—pyruvate, OAA—oxaloacetate, Pro—proline, NPT—non-protein thiols.

The results presented in the above subsections are summarized in Figure 6. In the leaf, most of the parameters studied remained unchanged or were upregulated in response to Ni treatment. The activities of NADH-GOGAT and NADH-GDH exhibited the most pronounced increases 2 and 3 weeks after the metal application, respectively. Decreases were observed in the case of Fd-GOGAT activity after 2 weeks of Ni exposure and in ${\mathrm{NO}}_{3}^{-}$ content after 2 and 3 weeks. In contrast to the leaf, most parameters in the root were not influenced by Ni stress or showed decreases, especially after 3 weeks. Only NADH-GDH and NAD-GDH activities were enhanced after Ni treatment.

### 2.7. N-Metabolism-Related Enzymes Gene Expression

**Figure 7:**
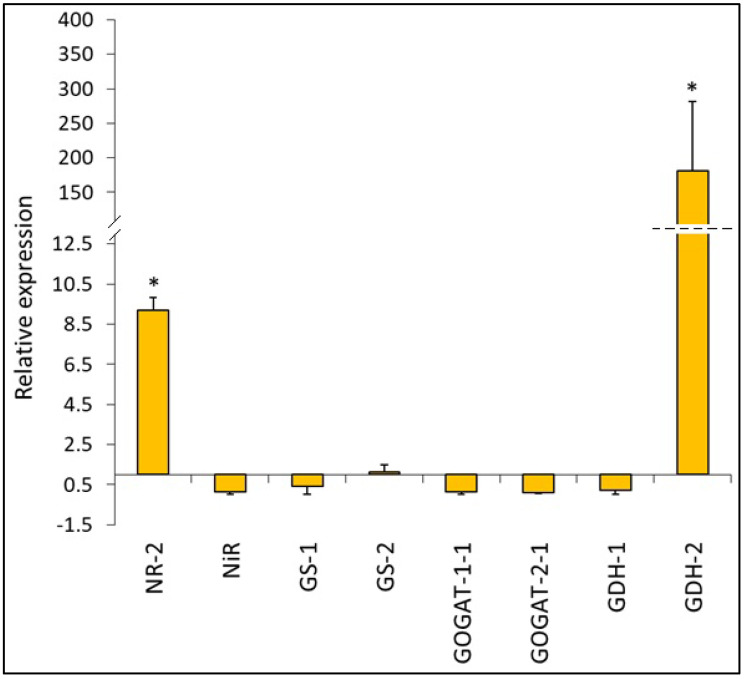
Relative expression of *NR-2*, *NiR*, *GS-1*, *GS-2*, *GOGAT-1-1*, *GOGAT-2-1*, *GDH-1*, and *GDH-2* in the cucumber leaf after 2 weeks of Ni treatment. Expression of genes relative to control plants (set to one) and normalized to two reference genes (*UBI-1*, *UBI-ep*) was calculated using REST. * indicates values that differ significantly from the control at *p* ˂ 0.05.

The expression of selected genes encoding N-metabolism-related enzymes was determined in the leaves 2 weeks after Ni application, and the results obtained are presented in Figure 7. Nickel treatment significantly upregulated *NR-2* gene expression, resulting in its 9-fold increase. On the contrary, *NiR* expression remained unaltered. Neither the expression of *GS1* encoding the cytosolic GS isoform nor the expression of *GS-2* encoding the chloroplastic isoform was significantly influenced by Ni stress. The expression of both *GOGAT-1-1* and *GOGAT-2-1* genes encoding Fd-GOGAT and NADH-GOGAT, respectively, showed a rather downward trend; however, the changes were not statistically significant. The most pronounced upregulation in response to Ni stress was observed in the case of *GDH-2*, which exhibited a 180-fold increase compared to the control. In contrast, the expression of *GDH-1* was not significantly altered.

## 3. Discussion

Supplementation of the nutrient medium with Ni led to a decrease in the ${\mathrm{NO}}_{3}^{-}$ content in the cucumber leaf; however, in the root it remained unaltered. Considering the previously reported data on the Ni influence on N transport in plants [1,2,3], it can be suggested that the observed decrease in this ion level in the leaf might be due to its impaired transport from the root to the aboveground part of the plant. Nickel-induced decline in the leaf ${\mathrm{NO}}_{3}^{-}$ level was also found in wheat [17], lettuce [19], and rice [20]. In contrast, an increase in ${\mathrm{NO}}_{3}^{-}$ content was detected in leaves of the Ni-treated sugarcane [21]. Despite a considerable decline in the ${\mathrm{NO}}_{3}^{-}$ content in the cucumber leaf, the ${\mathrm{NH}}_{4}^{+}$ level was unchanged. In the literature, both increases [20] and decreases [21] in the leaf ${\mathrm{NH}}_{4}^{+}$ content were reported for plants subjected to Ni stress.

Surprisingly, the reduction in ${\mathrm{NO}}_{3}^{-}$ content in the cucumber leaf was not accompanied by a decrease in NR activity. Although the ${\mathrm{NO}}_{3}^{-}$ supply is considered a limiting factor for this enzyme gene expression and activity [4,5], it seems that in the leaf of Ni-treated cucumber plants, this ion level was high enough to maintain the constitutive NR activity. Moreover, after 2 weeks of Ni exposure, we found a several-fold increase in *NR-2* gene expression. Since it was not followed by an increase in NR activity, some post-transcriptional or post-translational modifications might have occurred. Further study is needed to explain the discrepancy between *NR-2* gene expression and NR activity. Contrary to our result, a Ni-evoked decrease in NR activity was shown for sugar beet [22,23] and mustard [22,23] leaves, while in the sugarcane leaf, an induction of this enzyme activity was reported [21].

In contrast to the leaf, the cucumber root exhibited a considerable decrease in NR activity. Since no reduction in ${\mathrm{NO}}_{3}^{-}$ content was found in the root tissue, the limitation of substrate availability was not the reason for the lowered NR activity. Based on the literature data, it can be suggested that the observed decline in the root NR activity could rather be a consequence of the direct interaction of Ni with -SH groups of cysteine residues present in the enzyme. A limited supply of NADH was also proposed as an explanation for the inhibition of NR activity under heavy metal stress [4].

In our study, both in the leaf and the root, NiR activity was unaffected by Ni treatment. Nitrite reductase is considered to be more resistant to heavy metal stress than NR. This might be related to its localization in plastids, which hinders the metal approach to the enzyme [4]. Nonetheless, decreases in NiR activity were usually reported for Ni-stressed plants [17,18,20].

In line with the results of our earlier studies on wheat seedlings [17], the activity of GS in the cucumber leaf was not influenced by Ni application. On the contrary, the Ni-induced decline in this enzyme activity was found in the leaves of sugar beet [22] and rice [20]. The GS activity determined in our experiment was the sum of the cytosolic and the chloroplastic isoform activities. It has been suggested that a decrease in the activity of one isoform might be masked by an increase in another, resulting in an unchanged total GS activity [4]. Estimation of gene expression revealed that in the cucumber leaf, neither cytosolic nor chloroplastic GS gene expression was influenced by Ni stress. However, post-transcriptional regulation influencing the activity of GS isoforms cannot be excluded.

In contrast to the leaf, the cucumber root showed a decrease in GS activity. This could have resulted from a decline in the availability of ${\mathrm{NH}}_{4}^{+}$, whose level in the root tissues diminished in response to Ni treatment.

In our study, the activity of Fd-GOGAT, being the predominant form of GOGAT in leaves [4] decreased after the exposure of cucumber plants to Ni. To our knowledge, besides our earlier work demonstrating the Ni-evoked decline in Fd-GOGAT activity in the wheat shoot [17], there are no other reports on the Ni effect on this enzyme activity in plants. However, the negative impact of various heavy metals on Fd-GOGAT activity is well documented [4]. Contrary to the ferredoxin-dependent form, NADH-GOGAT, responsible for only 6% of the total GOGAT activity in cucumber leaf, was considerably increased in response to Ni treatment. A decrease in Fd-GOGAT activity, accompanied by an increase in the activity of NADH-GOGAT was previously observed in Ni-treated wheat shoots [17] and in the leaves of Cd-treated tomato [24]. Induction of NADH-GOGAT activity, probably showing lower sensitivity to heavy metals than Fd-GOGAT, may be a compensatory mechanism that enables the cyclic action of GS and GOGAT, allowing uninterrupted glutamate production.

Glutamate dehydrogenase, considered to play a negligible role in the primary N assimilation, seems to be an important source of glutamate under unfavorable environmental conditions, including heavy metal stress [4]. Plant GDHs exist in several isoenzymic forms, each being able to show both aminating (anabolic) and deaminating (catabolic) activity [4]. Both in the leaf and the root of the Ni-treated cucumber plants, we observed a marked increase in NADH-GDH activity. Increases in aminating GDH activity were also reported for plants treated with Cd [25], As [26], and Cu [27]. It can be suggested that the lack of ${\mathrm{NH}}_{4}^{+}$ accumulation in the tissues of Ni-stressed cucumber plants might be related to the intensive utilization of this ion in the NADH-GDH-catalyzed reaction.

Concomitantly with the increase in NADH-GDH activity, we observed an enhancement of the deaminating, NAD-dependent activity of this enzyme. A simultaneous increase in both NADH-GDH and NAD-GDH activities may reflect a dynamic turnover of glutamate in the tissues of Ni-treated cucumber plants. In the leaf, compared to NADH-GDH, the activity of NAD-GDH was less upregulated by Ni stress, which indicated that in this organ, the GDH-mediated glutamate turnover shifted towards the production of this amino acid. The parallel increase in NADH-GDH and NAD-GDH activities was previously reported for the roots of Cu-treated *Luffa cylindrica* [27]. An enhancement of GDH activity in the cucumber leaf was accompanied by a striking increase in *GDH-2* gene expression. According to the literature data [28] in plants, GDH is a hexamer formed from two main types of subunits: α and β. The α subunit is encoded by the *GDH-2* gene, while the β subunit by the *GDH-1* gene. These subunits combine in various ratios to create seven distinct NAD(H)-GDH isoenzymes. A marked increase in *GDH-2* gene expression found in the cucumber leaf may suggest an enhancement of α GHD subunits; however, explanation of the obtained results requires further study.

Our earlier study conducted on wheat seedlings revealed a Ni-induced increase in the shoot AlaAT and AspAT activities, suggesting involvement of these enzymes in glutamate production under stress conditions [17]. In contrast, in the cucumber leaves, aminotransferase activities were not influenced by Ni treatment, and in the roots, they were significantly downregulated.

The cytosolic NADP-isocitrate dehydrogenase is a key enzyme that links C and N metabolism by supplying 2-oxoglutarate for N assimilation [14]. We found a significant increase in this enzyme activity in the leaves of Ni-treated cucumber plants. Enhancement of NADP-ICDH under conditions of heavy metal stress was also reported for the leaves of Cd-treated pepper [29] and tomato [30] plants. León et al. [28] demonstrated that the Cd-induced increase in this enzyme activity was accompanied by an elevation of NADPH content. According to these authors, NADP-ICDH activity is a limiting factor in plant response to heavy metal stress. Another piece of evidence for the important role of NADP-ICDH in plant tolerance to heavy metals was provided by Liu et al. [31], who demonstrated that transgenic *Dianthus spiculifolius* plants overexpressing genes encoding this enzyme were more tolerant to Cd and Pb compared to the wild type. Increased NADP-ICDH activity in the tissues of *D. spiculifolius* mutant was accompanied by elevated content of 2-oxoglutarate. It has been suggested that 2-oxoglutarate, besides its role as a carbon skeleton in glutamate synthesis, might form stable chelates with heavy metal ions through its carboxylic groups [30].

Glutamate, the first organic product of N assimilation, is a multifunctional molecule. Apart from serving as a substrate for the production of other amino acids, it is also used in the synthesis of one involved in plant protection against stress [13]. Nickel did not influence glutamate content in the cucumber leaf, which is in contrast to our previous experiment on wheat [17] and the study of Lin and Kao [32], showing a decrease in this amino acid content.

Proline, a glutamate derivative, is primarily known for its osmoprotective properties and a crucial role in plant tolerance to salinity and drought. However, increases in free proline levels in plants exposed to heavy metals were also reported [33]. In line with other studies concerning Ni toxicity to plants [17,34,35,36], we observed an accumulation of proline in the cucumber leaf. The protective action of this amino acid under heavy metal stress was ascribed mainly to its functioning as a chelating and antioxidative agent. A relatively good stability of Ni-proline complexes was shown by Morzyk-Ociepa and Zelichowicz [37]. According to these authors, the chelating properties of proline are due to the presence of the ring N atom and COOH group in the molecule. It is possible that the increased proline level found in our experiment could be related to Ni chelation; however, further study involving, for example, spectroscopic methods, is needed to confirm the formation of Ni-proline complexes in cucumber tissues. The important role of proline in plant defense against heavy metal stress was confirmed by experiments with the exogenous use of this amino acid. Atta et al. [38] reported that the foliar application of proline markedly mitigated Ni-induced stress in wheat seedlings. Treatment with proline significantly improved the growth, photosynthesis, and antioxidant capacity of the Ni-stressed wheat. Accumulation of proline in plant tissues may result from its increased biosynthesis, decreased oxidation by proline dehydrogenase, or enhanced protein degradation [32]. Contrary to the findings of Lin and Kao [32] and Kevrešan et al. [22], who reported a Ni-induced decrease in protein content in rice and sugar beet leaves, respectively, the cucumber leaves responded to Ni treatment with a slight increase in total protein level. Therefore, enhanced proteolysis was not the reason for the enhancement of proline level in the cucumber leaf. It is believed that accumulation of this amino acid in response to heavy metal stress occurs mainly due to its increased synthesis via the glutamate pathway [36,39].

In contrast to the leaf, the root of the Ni-exposed cucumber plant showed a marked decrease in free proline level. Reduction in proline content was previously found in tomato plants treated with high concentrations of Cd, Cu, and Pb. It was associated with the negative effect of these metals on enzymes involved in proline biosynthesis [40]. It can be suggested that a decline in proline content in the cucumber root could be associated with the lowered level of glutamate, the main substrate for proline biosynthesis. Generally, it is considered that in heavy metal-treated plants, more proline is accumulated in the above-ground organs than in the roots. This may be related to the protection of the leaf tissues against dehydration, since water is indispensable for photosynthesis [33,36].

Exposure of cucumber plants to Ni led to a marked increase in non-protein thiol content in the leaf. Accordingly, a Ni-induced accumulation of these metabolites was observed in barley [41], rice [42], and *Brassica rapa* [43]. A study by Maleva et al. [44] confirmed the presence of Ni in the fraction of non-protein thiol compounds extracted from *Elodea canadensis* leaves. The pool of non-protein thiols comprises all low-molecular-weight compounds containing at least one sulfhydryl group (-SH) in their structure, including cysteine, glutathione, and phytochelatins [33,45,46]. Their role in plant response to heavy metal stress is related mainly to a high affinity of -SH groups for metal ions. Non-protein thiols bind heavy metals, preventing their interaction with cellular components. Among non-protein thiols, phytochelatins show the highest metal chelation ability. They are oligopeptides consisting of glutamate, cysteine, and glycine residues, synthesized from glutathione by phytochelatin synthase. Nickel is considered a poor inducer of phytochelatin synthesis and exhibits a relatively low affinity for -SH groups, compared to other heavy metals, especially Cd [33]. Nicotianamine, histidine, and carboxylic acids are believed to be much better Ni ligands than thiol compounds [1]. Nevertheless, accumulation of phytochelatins was found in the Ni-treated suspension-cultured tobacco cells [47]. Increased expression of the phytochelatin synthase gene was reported for Ni-exposed *Arabidopsis thaliana* [48] and forage pea [49]. It cannot be excluded that the accumulation of non-protein thiol compounds observed in the cucumber leaf might be, at least partly, due to elevated phytochelatin content.

Among the non-protein thiols, tripeptide glutathione (GSH, γ-glutamyl-cysteinyl-glycine) is the most abundant in plant cells [50]. Similarly to other molecules containing the -SH group, it can bind metals; however, it is primarily known for its antioxidant properties. Data concerning the Ni effect on GSH content in plants are contradictory; both its increases [23,35] and decreases [51] were reported. Apart from its direct reactive oxygen species scavenging activity, GSH also serves as a substrate for stress-related enzymes, such as glutathione S-transferase (GST), which catalyzes the conjugation of GSH to toxic lipophilic substrates, including aldehydes produced as a consequence of lipid peroxidation [45]. In our previous study, we found a 4-fold induction of GST activity in the cucumber leaf [52], indicating a high consumption of GSH by this enzyme.

## 4. Materials and Methods

### 4.1. Plant Material and Growth Conditions

Seeds of cucumber (*Cucumis sativus* L. cv. Cezar) were germinated for 6 days. Then the uniform seedlings were placed in Styrofoam plates (3 seedlings per plate) and transferred into plastic pots containing 1 dm^3^ of the diluted (1:4) Hoagland nutrient solution (composition of the nutrient solution is provided in Supplementary Materials in Table S1) containing 10 µM Ni supplied as chloride. An equimolar concentration of Cl^−^ (in the form of NaCl) was added to the control nutrient solution instead of NiCl_2_. The pH of the nutrient solution was adjusted to 5.8 and checked daily. The seedlings were grown hydroponically in a controlled climate room at 24 °C and 175 µmol m^−2^ s^−1^ photosynthetic photon flux density (PPFD), with 16 h photoperiod [52]. The nutrient solution was changed every 2nd day. After 2 and 3 weeks, the 1st (the oldest) leaves and roots were harvested. For estimation of Ni content, the leaves and roots were oven-dried. The other parameters were assayed in freshly harvested leaves and roots.

### 4.2. Growth Parameters and Ni Contents

Fresh weight (FW) of the leaves and roots were measured immediately after harvesting. Nickel contents in the cucumber tissues were determined by atomic absorption spectrometry. The dried tissue (100 mg) was digested in 5 cm^3^ 69% HNO_3_ using microwave mineralizer (UniClever, Plazmatronika, Wrocław, Poland). Nickel contents were determined using Varian SpectrAA 300 spectrometer (Varian Australia Pty. Ltd., Mulgrave, Australia) equipped with deuterium lamp for background correction and an air/acetylene flame at 232 nm [52]. Nickel contents in the leaves and roots were expressed in µg per g DW.

### 4.3. Nitrate, Ammonium, Glutamate, Proline, Non-Protein Thiols, and Total Protein Concentrations

For nitrate and ammonium estimation, fresh tissue was homogenized (1:10, *w*/*v*) in redistilled water, boiled for 15 min, and filtered.

Nitrate content was measured by the method of Cataldo et al. [53] based on the nitration of salicylic acid under acidic conditions. The reaction mixture consisted of 0.1 cm^3^ filtrate and 0.2 cm^3^ 5% (*w*/*v*) salicylic acid in concentrated H_2_SO_4_. After 15 min of incubation at room temperature, 1 cm^3^ 4M NaOH was added. After cooling the solution to room temperature, the absorbance was measured at 410 nm. Nitrate content was calculated using the calibration curve prepared for KNO_3_ and expressed as mg ${\mathrm{NO}}_{3}^{-}$ g^−1^ FW.

Ammonium content was determined colorimetrically using the Nessler reagent essentially as described by Molins-Legua et al. [54]. The reaction mixture consisted of 0.1 cm^3^ filtrate, 0.01 cm^3^ 10% (*w*/*v*) K–Na tartrate, 2.4 cm^3^ redistilled water, and 0.1 cm^3^ Nessler reagent. After 5 min, the absorbance was measured at 425 nm. Ammonium content was calculated using standard calibration curve prepared for NH_4_Cl and expressed as mg ${\mathrm{NH}}_{4}^{+}$ g^−1^ FW.

For estimation of glutamate content, fresh tissue was homogenized (1:10, *w*/*v*) in 0.05 M potassium phosphate buffer pH 7.0. After centrifugation (20,000× *g*, 20 min) in the supernatant, the obtained glutamate concentration was determined using a commercial enzymatic assay kit (Boehringer Mannheim/R-BIOPHARM, Darmstadt, Germany) and expressed in µmol g^−1^ FW.

Free proline content was determined using the ninhydrin method of Bates et al. [55]. Fresh tissue was homogenized (1:10, *v*/*w*) in 3% sulphosalicylic acid and centrifuged (18,000× *g*, 15 min). The supernatant obtained (1 cm^3^) was added to 1 cm^3^ glacial acetic acid and 1 cm^3^ acid ninhydrin. After boiling the mixture in a water bath at 100 °C for 60 min, the reaction was stopped by cooling the tubes in an ice bath for 5 min. The chromophore formed was extracted with 3 cm^3^ toluene and the tubes were placed in the dark for 50 min. Absorbance of the resulting organic layer was measured at 520 nm. The concentration of proline in the samples was estimated by referring to a standard curve for L-proline and expressed in nmol g^−1^ FW.

Non-protein thiol (NPT) content was assayed using 5,5′-dithiobis-(2-nitrobenzoic acid) (DTNB) according to Israr et al. [56]. Fresh tissue was homogenized (1:10, *w*/*v*) in a mortar with 5% sulphosalicylic acid and the homogenate was centrifuged at 20,000× *g* for 20 min. The assay mixture (1.65 cm^3^) consisted of the obtained supernatant, 0.1 M sodium phosphate buffer pH 7.0, 0.5 mM EDTA, and 0.25 mM DTNB. After 10 min of incubation at room temperature, the absorbance was measured at 412 nm. The concentration of NPT was estimated by referring to a standard curve prepared using GSH and expressed in nmol g^−1^ FW.

Total protein content was measured by the method of Bradford [57] with a standard calibration curve prepared using bovine serum albumin and expressed in mg g^−1^ FW.

### 4.4. Enzyme Extraction and Assays

#### 4.4.1. Nitrate Reductase and Nitrite Reductase

Fresh tissue was homogenized (1:5, *w*/*v*) in the ice-cold mortar using 0.1 M potassium phosphate buffer pH 7.5 containing 5 mM cysteine, 2 mM EDTA, and 0.5% (*w*/*v*) polyvinylpyrrolidone (PVP). After centrifugation (20,000× *g*, 20 min), the supernatant was used for the determination of NR and NiR activities.

NR activity was assayed according to the modified method described by Debouba et al. [58]. For the estimation of maximum NR activity, the reaction mixture (1.4 cm^3^) consisted of 0.1 M potassium phosphate buffer pH 7.5 containing 5 mM EDTA, 7 mM KNO_3_, 0.14 mM NADH, and enzyme extract. The reaction was started by the addition of NADH. After 30 min of incubation at 27 °C, the reaction was stopped by the addition of 0.1 cm^3^ 0.5 M zinc acetate and then the incubate was centrifuged (3000× *g*, 10 min). The nitrite formed was determined colorimetrically after diazotation with 1% (*w*/*v*) sulfanilamide (SA) and 0.01% (*w*/*v*) naphthylenediamine dihydrochloride (NEA). After 20 min of incubation at room temperature, the absorbance was measured at 540 nm and the amount of nitrite was calculated using standard calibration curve prepared for NaNO_2_. NR activity was expressed in nmol ${\mathrm{NO}}_{2}^{-}$ formed per min per mg protein.

NiR activity was measured as the reduction in the amount of ${\mathrm{NO}}_{2}^{-}$ in the reaction mixture according to the modified method of Debouba et al. [58]. The reaction mixture (2.5 cm^3^) consisted of 0.1 M potassium phosphate buffer pH 6.8, 0.4 mM NaNO_2_, 2.3 mM methyl viologen, enzyme extract, and 4.3 mM sodium dithionite in 100 mM NaHCO_3_, which started the reaction. After 30 min of incubation at 27 °C, the reaction was stopped by vortexing, and ${\mathrm{NO}}_{2}^{-}$ ions that remained in the reaction mixture were determined at 540 nm after reaction with SA and NEA as described above. NiR activity was expressed in nmol ${\mathrm{NO}}_{2}^{-}$ reduced per minute per mg protein.

#### 4.4.2. Glutamate Synthase

Fresh tissue was homogenized (1:5, *w*/*v*) in the ice-cold mortar using 50 mM potassium phosphate buffer pH 7.5 containing 2 mM EDTA, 10 mM KCl, 14 mM β–mercaptoethanol, 1 mM phenylmethylsulphonyl fluoride (PMSF), and 3.58 M ethylene glycol, and the homogenate obtained was centrifuged at 20,000× *g* for 20 min.

GOGAT activity was measured spectrophotometrically at 30 °C by monitoring the oxidation of NADH (ε = 6.22 mM^−1^cm^−1^) at 340 nm according to the modified method of Groat and Vance [59]. The reaction mixture (2 cm^3^) consisted of 0.1 M potassium phosphate buffer pH 7.5, 5 mM 2-oxoglutaric acid, 10 mM glutamine, 0.15 mM NADH, and enzyme extract. The enzyme activity was expressed in nmol NADH oxidation per minute per mg protein.

#### 4.4.3. Glutamine Synthetase, Glutamate Dehydrogenase, and Aminotransferases

Fresh tissue was homogenized (1:5, *w*/*v*) in the ice-cold mortar using 50 mM Tris–HCl buffer pH 7.6 containing 1 mM EDTA, 1 mM MgCl_2_, 10 mM β-mercaptoethanol, 1 mM dithiothreitol, and 0.5% (*w*/*v*) PVP. After centrifugation (20,000× *g*, 20 min), the supernatant was used for the determination of GS, NADH-GDH, NAD-GDH, AlaAT, and AspAT activities.

GS activity was estimated using the transferase assay, essentially as described by Agbaria et al. [60]. The reaction mixture (2 cm^3^) contained 50 mM Tris–HCl buffer pH 7.2, 1 mM ADP, 50 mM glutamine, 20 mM MgCl_2_, 20 mM sodium arsenate, enzyme extract, and 13 mM hydroxylamine. Addition of hydroxylamine started the reaction. After 30 min of incubation at 30 °C, the reaction was terminated by addition of 3 cm^3^ of the mixture consisting of 0.5 M HCl, 0.2 M FeCl_3_, and 0.24 M trichloroacetic acid. After centrifugation (3000× *g*, 10 min), the absorbance was measured at 540 nm. The enzyme activity was expressed in nmole γ-glutamylhydroxamate formed per minute per mg protein.

GDH activity was assayed spectrophotometrically at 30 °C by monitoring the oxidation of NADH (aminating GDH activity, NADH-GDH) or reduction in NAD (deaminating GDH activity, NAD-GDH) at 340 nm according to the method described by Groat and Vance [59]. For NADH-GDH activity, the reaction mixture (2 cm^3^) consisted of 0.1 M Tris–HCl buffer pH 8.0, enzyme extract, 11 mM 2-oxoglutaric acid, 0.1 M NH_4_Cl, and 0.2 mM NADH. For NAD-GDH activity, the reaction mixture (2 cm^3^) consisted of 0.1 M Tris–HCl buffer pH 8.8, enzyme extract, 80 mM L-glutamic acid, and 0.7 mM NAD. The enzyme activity was expressed in nmol NADH/NAD oxidized/reduced per minute per mg protein.

AlaAT and AspAT activities were assayed spectrophotometrically, essentially as described by Sousa and Sodek [61]. AlaAT activity was assayed in the alanine → pyruvate direction by coupling the reaction with NADH oxidation by lactate dehydrogenase. The reaction mixture (1.5 cm^3^) consisted of 0.1 M Tris–HCl buffer pH 7.5, 0.5 M L-alanine, 15 mM 2-oxoglutarate, 0.18 mM NADH, five units of lactate dehydrogenase, and enzyme extract. AspAT activity was assayed in the aspartate → oxaloacetate direction by coupling the reaction with NADH oxidation by malate dehydrogenase. The reaction mixture (1.5 cm^3^) consisted of 0.1 M Tris–HCl buffer pH 7.8, 5 mM EDTA, 0.2 M L-aspartate, 12 mM 2-oxoglutarate, 0.18 mM NADH, five units of malate dehydrogenase, and enzyme extract. In the case of both AlaAT and AspAT the reaction was conducted at 30 °C and the oxidation of NADH was monitored at 340 nm. AlaAT and AspAT activities were calculated using absorption coefficient for NADH (ε = 6.22 mM^−1^ cm^−1^) and expressed in nmole NADH oxidized per minute per mg protein.

#### 4.4.4. Isocitrate Dehydrogenase

Fresh tissue was homogenized (1:5, *w*/*v*) in a cooled mortar using 100 mM Tris–HCl buffer pH 8.0 containing 5 mM β-mercaptoethanol, 10% (*v*/*v*) glycerol, 1 mM EDTA, and 1 mM MgCl_2_, and the homogenate obtained was centrifuged at 20,000× *g* for 20 min.

NADP-ICDH activity was assayed by following NADP reduction according to Yang et al. [62]. The reaction mixture (1 cm^3^) consisted of 100 mM Tris–HCl buffer pH 8.0 containing 5 mM MgCl_2_, enzyme extract, 0.5 mM NADP, and 2.5 mM isocitric acid. For each sample, two blanks—lacking enzyme extract and lacking isocitric acid—were run simultaneously. NADP-ICDH activity was expressed in nmol NADPH formed per minute per mg protein.

### 4.5. Gene Expression Analysis

The gene expression profile was analyzed in leaf of cucumber seedlings after 2 weeks of Ni treatment.

Total RNA was extracted using Bluezol (Serva Electrophoresis GmbH, Heidelberg, Germany) following the manufacturer’s instructions. RNA precipitation step was modified to remove polysaccharide contamination and 0.25 cm^3^ of isopropanol followed by 0.25 cm^3^ of a high salt precipitation solution (0.8 M sodium citrate and 1.2 M NaCl) per 1 cm^3^ of Bluezol was added to the aqueous phase. The total RNA was quantified by spectrophotometric assay, and the quality of isolated RNA was evaluated by gel electrophoresis. Intact rRNA subunits of 28S and 18S were observed on the gel which indicated minimal degradation of the RNA. Thereafter, 1 μg of total RNA was reverse transcribed using High-Capacity cDNA Reverse Transcription Kit (Applied Biosystems, Waltham, MA, USA) following manufacturer’s recommendations.

Quantitative Real-time PCR assays were performed on the 7500 Real Time PCR System (Applied Biosystems). Each 10 μL reaction was carried out in a reaction mixture containing the following: 1× concentrated Real-Time 2xHS-PCR Master Mix SYBR A (A&A Biotechnology, Gdańsk, Poland) including LoROX fluorochrome internal control, 2.5 μM specific primers (Supplementary Table S2), cDNA template (in three concentrations, each in duplicate), and water. The cycling profile consisted of an initial cycle at 95 °C for 5 min, followed by 45 cycles of denaturation (95 °C for 15 s) and primer annealing-extension (60 °C for 1 min). After PCR cycling, melting curve analysis was performed in the range of 60–95 °C.

Relative Expression Software Tool (REST, 2009) was used to calculate relative *NR-2*, *NiR*, *GS-1*, *GS-2*, *GOGAT-1-1*, *GOGAT-2-1*, *GDH-1*, and *GDH-2* gene expression by the ΔCt method with geometric mean of two reference genes: *UBI-1* and *UBI-ep*.

### 4.6. Statistical Analysis

The results are the means of three independent experiments. For fresh weight estimation, 3–6 plants were analyzed per treatment in each experiment, so the data present the means of 9–18 observations. For the other parameters, 1–3 samples were analyzed per treatment in each experiment (*n* = 3–9). The sample for analysis was taken from a single plant. The significance of differences between Ni-treated seedlings and the control was estimated using one-way ANOVA followed by Fisher’s least significant difference (LSD) test (*p* < 0.05). Sample variability was given as the standard deviation of the mean. The statistical analysis was carried out using Statistica 13.1 software.

## 5. Conclusions

In conclusion, the results of our study indicate the Ni-induced disturbance in primary N assimilation in the cucumber plant. In the root, except for an increase in GDH activity, N metabolism was negatively influenced by Ni stress, which was reflected by decreased glutamate, proline, and non-protein thiol contents. In the leaf, increases in proline and non-protein thiol contents in response to Ni application implied the existence of a large demand for glutamate. Accumulation of these protective metabolites without significant changes in glutamate content may suggest that in the leaf, the induction of NADH-GOGAT and NADH-GDH activities efficiently compensates for the reduced Fd-GOGAT activity. Additionally, the increased NADP-ICDH activity may support glutamate production by providing 2-oxoglutarate for reactions catalyzed by NADH-GOGAT and NADH-GDH. The distinctive response of the cucumber leaf and root to Ni exposure may be related to the differential accumulation of the metal in these organs. Our study provided interesting findings concerning the Ni effect on nitrogen metabolism in cucumber plants; however, further study is needed to explain some of the results we obtained. Considering the marked discrepancies found between Ni-induced changes in gene expression levels and enzyme activities, future research should focus on elucidating the post-transcriptional and post-translational mechanisms of enzyme protein modification.

## Figures and Tables

**Table 1:** Effect of Ni treatment on growth, Ni accumulation in the cucumber leaf and root. Data represent mean values ± SD (*n* = 4–18); * indicates values that differ significantly from the control at *p* < 0.05.

Treatment		Fresh Weight [mg]		Ni Content [μg g ^−1^ DW]	
		**Week 2**	**Week 3**	**Week 2**	**Week 3**
Leaf	Control	284.3 ± 58.9	360 ± 55.33	1.98 ± 0.75	2.29 ± 0.25
	Ni	133.6 ± 43.3 *	214.23 ± 39.68 *	292.85 ± 65.81 *	286.45 ± 41.87 *
Root	Control	533.47 ± 104.87	927.92 ± 260.7	4.38 ± 0.42	3.25 ± 0.44
	Ni	221.24 ± 58.77 *	465.11 ± 167.58 *	592.89 ± 82.12 *	502.28 ± 194.27 *

## Data Availability

The original data presented in the study are openly available in [Effect of nickel stress on nitrogen metabolism in cucumber plants_Manuscript data] at [https://uniwersytetlodzki-my.sharepoint.com/:x:/g/personal/ewa_gajewska_biol_uni_lodz_pl/ETOh6DA0P1tNqweCgM-v1ugBNLR_9JdeZ0Gqsv_volaxRA?e=iJaHrb, accessed on 30 July 2025].

## Supplementary Materials

The following supporting information can be downloaded at https://www.mdpi.com/article/10.3390/ijms26199327/s1, Ref. [63] is cited in the Supplementary Materials.

## Abbreviations

Ala	Alanine
AlaAT	Alanine aminotransferase
Asp	Aspartate
AspAT	Aspartate aminotransferase
DTNB	5,5′-dithiobis-(2-nitrobenzoic acid)
DW	Dry weight
EDTA	Ethylenediaminetetraacetic acid
FW	Fresh weight
GDH	Glutamate dehydrogenase
Gln	Glutamine
Glu	Glutamate
GOGAT	Glutamate synthase
GS	Glutamine synthetase
GSH	Glutathione
NADP-ICDH	Isocitrate dehydrogenase
NEA	Naphthylenediamine dihydrochloride
NiR	Nitrite reductase
NPT	Non-protein thiols
NR	Nitrate reductase
OAA	Oxaloacetate
2-OG	2-Oxoglutarate
PMSF	Phenylmethylsulphonyl fluoride
Pro	Proline
PVP	Polyvinylpyrrolidone
PYR	Pyruvate
SA	Sulfanilamide
