# Supplementary material for: Effect of Nickel Stress on Nitrogen Metabolism in Cucumber Plants
Source: Int J Mol Sci. 2025 Sep 24;26(19):9327. doi: 10.3390/ijms26199327 (PMC12524678; doi:10.3390/ijms26199327)
Supplement: Supplementary file 1 [file ijms-26-09327-s001.zip › ijms-3822971-supplementary.pdf]

Supplementary Table S1. Composition of the diluted Hoagland nutrient solution used for cucumber hydroponic growing.

Compound	Content [mg dm^{-3}]
$Ca(NO_3)_2 \times 4 H_2O$	295
KNO_3	126.25
KH_2PO_4	34
$MgSO_4 \times 7 H_2O$	123
FeNaEDTA	10.0775
$MnSO_4 \times 5 H_2O$	0.5475
H_3BO_3	0.715
$CuSO_4 \times 5 H_2O$	0.01975
$ZnSO_4 \times 7 H_2O$	0.55
$Na_2MoO_4 \times 2 H_2O$	0.0325

Supplementary Table S2. Primer sequences for target and reference genes [63]

NCBI Accession number	Biological Process and Molecular Function	Forward Primer	Reverse Primer
**NM001280767.1 (NR-2)**	Nitrate reductase [NADH]-like (NR2)	TACTGGTGCTGGTGTTTCTGGTC	GATTTCTCCCTTGTGAGGTTTGC
**XM004140647.2 (NiR)**	Ferredoxin-nitrite reductase, chloroplastic (NIR)	GTCCCTCTCTGTGGAGCCATCTT	CCCTTCTTTCCCATTGCTTATTT
**NM001280715.1 (GS-1)**	Glutamine synthetase cytosolic isozyme-like (GS1)	TTCTTTCTTTTGATCCAAAACCA	ATGTCGCCCTGTGAGACGACGCT
**XM004134113.2 (GS-2)**	Glutamine synthetase leaf isozyme, chloroplastic transcript variant X1	GTGCCCATCCCTACAAACAAACG	ACACCACAGTAATAAGGCCCCTG
**XM004136730.2 (GOGAT-1-1)**	Ferredoxin-dependent glutamate synthase, chloroplastic, transcript variant X1	GAACGAGAACTTTACATTTGTAG	CTATATCTTCGATGATAAATAGC
**XM011653296.1 (GOGAT-2-1)**	Glutamate synthase [NADH], amyloplastic, transcript variant X1	AGTTGGGATCGTGCTCAGCCT	CTAATTAAAAGCTCAAGAACACC
**XM004147487.2 (GDH-1)**	Glutamate dehydrogenase 1, transcript variant X2	GCAATCCTGGAGAATTAAGTATA	AGAGATCCACCTAGATCAATAGG
**XM004146845.2 (GDH-2)**	Glutamate dehydrogenase 2	TAAAGAAAGTACTGGAAGCCTTG	TCATCTGCCTCTGGATCTGTGGG
**AF104391.1 (UBI-1)**	Ubiquitin-like protein	CCTTATTGACCAACCAGTAGT	GGACAATGTTGATTTCCTCG
**AY372537.1 (UBI-ep)**	Ubiquitin extension protein	CACCAAGCCCAAGAAGATC	TAAACCTAATCACCACCAGC
